# Impact of different forest types on soil microbial biomass and microbial entropy in the karst region of southwestern China

**DOI:** 10.3389/fpls.2025.1678667

**Published:** 2025-11-03

**Authors:** Yan Wu, Xiaoli Zhong, Xun Liu, Bo Ding, Yunlin Zhang

**Affiliations:** ^1^ College of Biological Sciences, Guizhou Education University, Guiyang, China; ^2^ Key Laboratory of Forest Fire Ecology and Management, Guizhou Provincial Department of Education, Guiyang, China

**Keywords:** karst, forest type, soil microbial biomass, soil microbial entropy, ecological stoichiometry characteristics

## Abstract

Soil microbial biomass and microbial entropy are used as important indicators of soil quality. However, the effects of forest-stand types remain poorly understood. This study focused on three stands of Cryptomeria japonica var. sinensis (CJ), Liquidambar formosana (LF), and their mixed forests (CL) in Guizhou Province, China. Soil samples were collected from three depths to investigate variations in soil microbial biomass C, N, P (MBC, MBN, MBP), as well as microbial entropy C, N, P (qMBC, qMBN, qMBP) among different forest stands. Additionally, the influence of soil organic C (SOC), total N (TN), total P (TP), and their stoichiometry, along with soil microbial C:N:P stoichiometry and soil-microbial stoichiometric imbalances on soil microbial biomass and microbial entropy are analyzed. The variance analysis revealed, compared to pure stands, the mixed forest exhibited significantly higher MBC (38.84%), MBC stocks (46.72%), MBC/MBN (52.23%), MBC/MBP (52.23%), and qMBC (23.49%; *p* < 0.05). Pure stand LF showed approximately 30% higher soil microbial stoichiometric imbalances (C/N_imb_, C/P_imb_, and N/P_imb_) than the other two stand types (*p* < 0.05). While the pure CJ stand exhibited significantly higher qMBN and qMBP (19.62% and 17.26%, respectively; *p* < 0.05). MBC, MBN, MBP, and their storage decreased significantly with increasing soil depth (*p* < 0.05), no significant effect on microbial stoichiometric ratios or microbial entropy. Correlation and redundancy analyses demonstrated that MBC, MBN, and MBP were highly significantly positively correlated with SOC, TN, and TP contents (*p* < 0.01), whereas qMBC and qMBN exhibited highly significant negative correlations with SOC, TP, SOC/TP, TN/TP, C/P_imb_, and N/P_imb_ (*p* < 0.01). Soil TP and MBC/MBP were identified as the primary factors influencing soil microbial biomass variation, with explanatory rates of 42.8% and 14.8%, respectively. Furthermore, C/N_imb_ and C/P_imb_ emerged as key determinants affecting microbial entropy dynamics, accounting for 31.5% and 14.2% of the observed variation, respectively. This study provided valuable data and insights for developing mixed forest management strategies in karst areas.

## 1 Introduction

As core drivers of soil ecosystem functions, soil microorganisms play crucial roles in decomposing organic matter, regulating nutrient cycles, storing C, maintaining soil productivity, and determining soil fertility (Hartmann and Six, 2023). Soil microbial biomass refers to the total biomass of living components of soil organic matter with a volume smaller than 5.0×10³ μm³, primarily including bacteria, fungi, algae, and protozoa (Li et al., 2018). Its content reflects the mineralization capacity of the soil and soil vitality, serving as an important parameter for characterizing material cycles and energy flows in soil ecosystems. MBC, MBN and MBP are highly sensitive to environmental changes and exhibit rapid turnover rates, making them common indicators for assessing changes in soil fertility and soil quality (Song et al., 2022; G. H. Zhang et al., 2023). Soil microbial entropy refers to the proportion of microbial biomass C, N, and P in soil organic C (SOC), total N (TN), and total P (TP), respectively. It effectively measures the level of microbial biomass sustained per unit resource (Heuck et al., 2015). Owing to its high sensitivity to nutrient utilization efficiency, it serves as a reliable biological indicator for evaluating dynamic changes in soil ecosystems and can also reflect the evolution of soil quality and the characteristics of nutrient accumulation (Ji et al., 2020).

The soil-microbial stoichiometric imbalance measures the discrepancy between microbial biomass and the chemical composition of soil resources. A smaller value indicates higher soil resource quality and greater microbial growth efficiency, helping to clarify the dynamic nutrient balance between the soil and microorganisms (Mooshammer et al., 2014). Research on the ecological stoichiometry of soil microbial C, N, and P can enhance our understanding of microbial ecological processes and their underlying mechanisms (X. Z. Wu et al., 2019). Since soil C:N:P ratios significantly influence microbial community structure, biomass, and microbial entropy, elucidating the relationships among soil microbial biomass, microbial entropy, soil C-N-P stoichiometry (Zechmeister-Boltenstern et al., 2015; Zhou and Wang, 2016), and soil-microbial stoichiometric imbalance is crucial for uncovering the mechanisms of soil nutrient balance (Zhang et al., 2019).

Forest ecosystems are the core of Earth’s life-support system and play a vital role in climate regulation, biodiversity conservation, water retention, and environmental stability (Spiridonov et al., 2025). Soil microorganisms act as engines for forest health and productivity by facilitating nutrient cycling, maintaining soil structure, and enhancing plant stress resistance through processes such as N fixation, P solubilization, soil particle aggregation via hyphae and secretions, and symbiotic relationships with plants via mycorrhizal fungi (Hu et al., 2024). Extensive research has been conducted on soil microbial biomass and its stoichiometric characteristics in forest ecosystems, with focus on various factors, including climate change (Tian et al., 2023), litter input (Jing et al., 2021), forest fires (Singh et al., 2021), altitude (R. Q. Wang et al., 2024), land-use changes (Jiang et al., 2024), and tree species composition (Babur et al., 2021).

Specifically, the influence of tree species on soil microbial biomass has garnered significant attention. The tree species composition fundamentally determines forest ecosystem characteristics (Shi et al., 2022). Furthermore, distinct stand types composed of different tree species significantly alter soil microbial communities through variations in root exudates, quantity and quality of litter/root inputs, and differences in litter decomposition rates and growth patterns (Song et al., 2020). However, because of species-specific nutrient acquisition strategies that substantially affect litter production, chemical composition, and decomposition dynamics, comparative studies of soil microbial biomass across various stand types have yielded inconsistent results (Siwach et al., 2024). Significant differences were observed in the soil microbial biomass and community structure between *Pinus massoniana* forests and citrus plantations on Jinyun Mountain (Zeng et al., 2015). Studies demonstrated that in subtropical mature forests, broad-leaved forests exhibited a higher MBP content than coniferous and mixed coniferous broad-leaved forests (Hou et al., 2014). In the Central Himalayas, a study of five coniferous and broad-leaved forest types revealed that oak and deodar cedar forests contain the highest levels of MBC, MBN, and MBP (Siwach et al., 2024). The study of pine camphor mixed forests and monocultures revealed that mixed forests had higher soil MBC and MBN values than pure forests (Dong et al., 2017). Xu et al. (2014) investigated the effects of vegetation on soil microbial biomass and observed that microbial biomass varied significantly among tree species and was negatively correlated with the soil C:N ratio.

Current research on soil microbial entropy remains primarily focused on its responses to factors such as land-use patterns (Chi et al., 2023), farming practices in agricultural ecosystems (Li et al., 2024), afforestation in severely degraded areas (Liu et al., 2012), and tea plantation chronosequences (G. H. Zhang et al., 2023). However, studies on how forest stand type affects soil microbial entropy and its driving factors are lacking. Xu et al. (2014) identified soil temperature, moisture, and substrate quality as key regulators of microbial entropy. Hu et al. (2021) investigated the subalpine natural secondary forests of western Sichuan, finding that qMBC decreased with increasing soil N/P ratio, whereas qMBP showed positive correlations with both soil C/P and N/P ratios. These findings suggest that future studies on stand-type effects on soil microbial biomass and entropy should incorporate comprehensive analyses that consider regional variations, soil types, and tree species characteristics.

Karst landforms are widespread globally, covering approximately 12–15% of the Earth’s terrestrial surface. Karst regions in China are predominantly distributed in the southwest, spanning approximately 550,000 km², making it one of the world’s three largest concentrated karst distribution areas (Zhang et al., 2022). Guizhou Province, located at the core of the ecologically fragile southwest karst region, has exposed and covered karst areas that account for 73.8% of the total land area (Zhong et al., 2021). This region’s unique eco-geological environment contributes to its ecological vulnerability, which is characterized by high ecological sensitivity, low environmental carrying capacity, weak disturbance resistance, and poor stability (Peng et al., 2023). Since the 1990s, China has implemented comprehensive national forestry projects in the southwest karst region, including the Grain-for-Green Program, Natural Forest Protection Program, and Shelterbelt Development Program, to address rocky desertification. By the end of 2023, the province’s forest area reached 11.1 million ha, with a forest coverage rate of 63%. *Cryptomeria japonica* var. *sinensis* (CJ), pure stands of *Liquidambar formosana* (LF) are pioneering tree species for ecological restoration in karst areas. The afforestation area of CJ forest is 143800 hectares, and the afforestation area of LF forest is 117200 hectares, accounting for 2.2% and 1.8% of the total area of tree forests in Guizhou Province (6.68 million hectares), respectively (Zhang and Ding, 2019; Zhang and Guo, 2020). Currently, the mixed afforestation model of CJ and LF has been successfully implemented in the Zhazuo Experimental Forest Farm, optimizing the forest structure. Previous studies have focused on afforestation in karst areas, which has improved soil physicochemical properties (Jian et al., 2006), promoted the accumulation of glomalin-related soil protein (Ling et al., 2025), enhanced soil carbon sequestration and water retention capacity (Pang et al., 2025), and altered the migration characteristics of nitrogen and phosphorus nutrients (Zheng et al., 2023). Some scholars have also found that mixed forests show higher shrub-layer species richness and greater beta diversity than pure forests (Kumilamba et al., 2025). However, research on how different forest stand types affect soil microbial biomass and microbial quotient under similar site conditions, stand characteristics, and management histories in the karst areas of Guizhou remains limited.

To address these limitations, this study investigated three forest stand types in the Zhazuo Experimental Forest Farm in Guizhou Province: pure stands of *Cryptomeria japonica* var. *sinensis* (CJ), pure stands of *Liquidambar formosana* (LF), and mixed stands of *Cryptomeria japonica* var. *sinensis* and *Liquidambar formosana* (CL). This study aimed to (1) investigate the variation patterns of soil microbial biomass, microbial entropy, and stoichiometric characteristics across different forest types and (2) analyze the influences of soil C-N-P stoichiometry, microbial stoichiometric ratios, and soil-microbial stoichiometric imbalance on soil microbial biomass and entropy. These findings provide theoretical references and fundamental data for supporting forest ecological restoration and sustainable development in karst regions.

## 2 Materials and methods

### 2.1 Overview of the study area

The study area is located in Zhazuo Forest Farm, Xiuwen County, Guiyang City, Guizhou Province, (106°36’–107°3’E and 26°2’–26°59’N), with a total operating area of 10786.73 hm^2^, of which the forest ecosystem occupies a dominant position and the vegetation coverage is 89.21%. The average altitude is 1290m, which corresponds to a typical karst plateau area. The climate type is subtropical monsoon humid climate, with an average annual temperature of 12.8–14.6°C, rainfall of 877–1121 mm, 1300 average sunshine hours, and frost-free period of 261 d (Wang et al., 2023). The soil types were mostly acidic or slightly acidic yellow loam. The vegetation types of the forest farm were mostly artificial vegetation. The trees mainly included *Cryptomeria japonica* var. *Sinensis* Miq., *Pinus massoniana* Lamb., and *Liquidambar formosana* Hance. The shrubs mainly included *Serissa serissoides* (DC.) Druce, *Viburnum dilatatum* Thunb., and *Corylus heterophylla* Fisch. exTrautv. The herbs mainly included *Hypolepis punctata* (Thunb.) Mett., *Diplazium donianum* (Mett.) Tardieu, and *Ophiopogon bodinieri* H. Lév.

### 2.2 Plot setting and sample collection

Three typical stand types (CJ, LF, and mixed (CJ×LF)) were selected from a forest farm, and sample plots were constructed based on the principle of consistency of topography, altitude, slope, slope aspect, and forest age. Three replicate plots were established for each stand type, with nine standard plots established. The plots were set up in 20 × 20 m (400 m^2^) squares and the DBH, tree height, and stand density of the trees in the tree layer were investigated. Basic information on the sampling sites is presented in **Table 1**.

**Table 1:** Basic characteristics of sample plots in the study area.

Sample plot	Forest types	Latitude and longitude	Forest age	Altitude(m)	Slope(°)	Average TH(m)	Average DBH (cm)	Stand density (plant/hm^2^)
1	CJ	106°41’ 28.68”E 26°49’ 16.68”N	21	1333	55	17.71	21.72	625
2	CJ	106°42’ 33.12”E 26°45’ 33.84”N	21	1349	45	16.77	23.6	725
3	CJ	106°43’ 10.92”E 26°43’ 13.08”N	23	1323	54	16.54	23.94	850
4	LF	106°43’ 09.84”E 26°53’ 13.92”N	22	1317	53	18.14	26.81	650
5	LF	106°42’ 11.88”E 26°46’ 16.68”N	21	1317	48	15.97	27.98	725
6	LF	106°40’ 30.72”E 26°46’ 10.92”N	21	1317	60.5	16.62	28.25	750
7	CL	103°40’ 13.92”E 26°51’ 07.92”N	22	1337	58.5	19.32	26.58	550 CJ:40%; LF: 56%
8	CL	106°40’ 30.72”E 26°46’ 30.72”N	21	1339	53.1	15.59	30.97	475 CJ:55%; LF: 36%
9	CL	106°43’ 06.96”E 26°45’ 30.96”N	22	1350	40	16.06	22.71	525 CJ:42%; LF: 45%

CJ is the abbreviation for pure stands of *Cryptomeria japonica* var. *sinensis*; LF is the abbreviation for pure stands of *Liquidambar formosana*; CL is the abbreviation for mixed stands of *Cryptomeria japonica* var. *sinensis* and *Liquidambar formosana*.

In the sample plot, the “S” type sampling method was used. Soil samples at 0–20 cm, 20–40 cm, and 40–60 cm depth were collected using a ring knife at 5 points and impurities such as gravel, plant roots, and animal remains were manually removed. The samples were passed through a 2-mm pore size sieve and stored at 4°C for soil microbial biomass determination.

### 2.3 Sample determination

Quantitative analysis of C, N, and P in the soil microbial biomass was performed using the chloroform fumigation-K_2_SO_4_/NaHCO_3_ extraction method based on the principle of biomass intracellular material release. SOC, TN, and TP in the extract were detected using the potassium dichromate external heating, semi-trace Kjeldahl determination, and molybdenum-antimony anti-colorimetric methods, respectively. The MBC, MBN, and MBP contents were calculated using the following formulas (Wu et al., 2006):


$$\text{MBC}=\text{EC}/k_{\text{EC}}$$

$$\text{MBN}=\text{EN}/k_{\text{EN}}$$

$$\text{MBP}=\text{EPt}/k_{\text{P}}$$

where EC, EN, and EPt are the differences between the SOC, TN, and TP of the fumigated and unfumigated soils, respectively, and *k*_EC_, *k*_EN_, and *k*_P_ are the conversion coefficients of MBC, MBN, and MBP, with values of 0.45, 0.54, and 0.40, respectively.

### 2.4 Data analysis

The formulae for calculating the soil microbial biomass C, N, and P storage (Mg/hm^2^), soil microbial entropy, and soil-microbial stoichiometric imbalance are as follows (Chi et al., 2023):


Soil microbial biomass C stocks(MBCS)=MBC×SBD×D/10000

Soil microbial biomass N stocks(MBNS)=MBN×SBD×D/10000

Soil microbial biomass P stocks(MBPS)=MBP×SBD×D/10000

Soil microbial entropy C(qMBC)=MBC/SOC×100%

Soil microbial entropy N(qMBN)=MBN/TN×100%

Soil microbial entropy P(qMBP)=MBP/TP×100%

$$\text{C}/\text{N stoichiometry imbalance}(\text{C}/{\text{N}}_{\text{imb}})=\text{SOC}:\text{TN}/\text{MBC}:\text{MBN}$$

$$\text{C}/\text{P stoichiometry imbalance}(\text{C}/{\text{P}}_{\text{imb}})=\text{SOC}:\text{TP}/\text{MBC}:\text{MBP}$$

$$\text{N}/\text{P stoichiometry imbalance}(\text{N}/{\text{P}}_{\text{imb}})=\text{TN}:\text{TP}/\text{MBN}:\text{MBP}$$

where SBD is the bulk density of the soil (g/cm^3^) and D is the thickness of the soil layer (cm).

Excel is used for data organization. A one-way variance model was constructed by SPSS 28.0 statistical software, and multiple comparisons (significance threshold α=0.05) were used to evaluate the differences of indicators among different forest types. Simultaneously, a bivariate correlation matrix was constructed, and the degree of correlation between the parameters was investigated based on the Pearson correlation analysis. Origin 2024 software was used to complete the drawing of various statistical charts. Redundancy analysis and mapping were performed using Canoco5 software to explore the effects of soil microbial and stoichiometric imbalances on soil microbial biomass and microbial entropy.

## 3 Results

### 3.1 Soil microbial biomass, storage, and stoichiometry across different stand types

As shown in **Table 2**, the contents of MBC, MBN, and MBP ranged from 1.69–4.68, 0.06–0.17, and 0.05–0.13 g/kg, respectively, while their storage levels ranged from 3.64–9.12, 0.13–0.33, and 0.11–0.24 Mg/hm². Aggregated data (0–60 cm) revealed that the mixed forests had significantly higher MBC and storage than the pure stands (CJ and LF; *p* < 0.05). Specifically, in the 20–40 and 40–60 cm soil layers, mixed forests exhibited 52.63–54.44% higher MBC content and 45.13–66.76% higher MBC storage compared to CJ and LF (*p* < 0.05). For MBN and MBP, no significant differences were observed among the three stand types at 0–60 cm depth (*p* > 0.05). However, in the 20–40 cm layer, LF exhibited significantly higher MBN content and storage than CJ mixed forests (*p* < 0.05). Meanwhile, MBP content and storage followed different trends across soil layers, showing distinct heterogeneity: in the 0–20 cm layer, CJ and mixed forests had significantly higher MBP; in the 20–40 cm layer, CJ showed significantly higher MBP; and in the 40–60 cm layer, CJ and LF showed significantly higher MBP (*p* < 0.05).

**Table 2:** MBC, MBN, MBP and their storage among different forest types.

Index	Forest types	Soil layers(cm)			
		0-20	20-40	40-60	0-60
MBC(g/kg)	CJ	3.85 ± 0.21Aa	2.47 ± 0.14Bb	1.69 ± 0.14Bc	2.67 ± 0.18B
	LF	3.81 ± 0.28Aa	2.47 ± 0.14Bb	1.71 ± 0.26Bc	2.66 ± 0.18B
	CL	4.68 ± 0.49Aa	3.81 ± 0.38Aab	2.61 ± 0.34Ab	3.70 ± 0.27A
MBN(g/kg)	CJ	0.17 ± 0.00Aa	0.10 ± 0.01Bb	0.06 ± 0.00Ac	0.11 ± 0.01A
	LF	0.17 ± 0.01Aa	0.12 ± 0.00Ab	0.07 ± 0.00Ac	0.12 ± 0.01A
	CL	0.14 ± 0.01Ba	0.10 ± 0.01Bb	0.06 ± 0.00Ac	0.10 ± 0.01A
MBP(g/kg)	CJ	0.13 ± 0.01Aa	0.11 ± 0.01Ab	0.06 ± 0.01Ac	0.10 ± 0.01A
	LF	0.11 ± 0.00Ba	0.09 ± 0.00Bb	0.07 ± 0.00Ac	0.09 ± 0.00A
	CL	0.13 ± 0.01Aa	0.07 ± 0.00Bb	0.05 ± 0.00Bc	0.09 ± 0.01A
MBCS(Mg/hm^2^)	CJ	7.04 ± 0.39Ba	5.11 ± 0.29Bb	3.64 ± 0.33Bc	8.47 ± 0.49B
	LF	7.90 ± 0.54ABa	5.54 ± 1.02Bb	3.80 ± 0.11Bc	8.85 ± 0.55B
	CL	9.12 ± 2.64Aa	8.04 ± 0.79Aab	6.07 ± 0.82Ab	12.47 ± 0.91A
MBNS(Mg/hm^2^)	CJ	0.31 ± 0.01Aa	0.21 ± 0.02Bb	0.13 ± 0.01Ac	0.35 ± 0.02A
	LF	0.31 ± 0.01Aa	0.25 ± 0.01Ab	0.15 ± 0.01Ac	0.40 ± 0.02A
	CL	0.33 ± 0.04Aa	0.20 ± 0.01Bb	0.14 ± 0.01Ac	0.35 ± 0.02A
MBPS(Mg/hm^2^)	CJ	0.23 ± 0.01Aa	0.22 ± 0.01Aa	0.13 ± 0.01Ab	0.32 ± 0.02A
	LF	0.20 ± 0.00Ba	0.18 ± 0.00Bb	0.15 ± 0.01Ac	0.29 ± 0.01A
	CL	0.24 ± 0.01Aa	0.16 ± 0.01Bb	0.11 ± 0.00Bc	0.29 ± 0.02A

The data is mean ± standard error; Different capital letters indicate significant differences (*p* < 0.05) among different forest types in the same soil layer; Different lowercase letters indicate significant differences (*p* < 0.05) among different soil layers in the same forest type. Same below. MBC, MBN and MBP are the abbreviation for soil microbial biomass C, microbial biomass N and microbial biomass P, respectively; MBCS, MBNS and MBPS are the abbreviation for soil microbial biomass C stocks, microbial biomass N stocks and microbial biomass P stocks, respectively.

**Table 3** shows that stand type had a significant effect on soil microbial stoichiometric characteristics. The aggregated data (0–60 cm) showed that the mixed forests had significantly higher MBC/MBN and MBC/MBP ratios than CJ and LF. The MBC/MBN ratio followed a consistent trend across 0–20, 20–40, and 40–60 cm soil layers, with mixed forests exhibiting 1.41–1.81 times higher values than CJ and LF (*p* < 0.05). The MBC/MBP ratio was significantly higher in mixed forests by 81.90–124.01%, particularly in the subsurface (20–40 cm) and deep soil layers (40–60 cm) (*p* < 0.05). For the MBN/MBP ratio, the 0–60 cm data revealed that LF had significantly higher values than CJ, whereas mixed forests showed no significant differences compared to CJ or LF. However, the MBN/MBP ratio exhibited heterogeneous patterns across soil layers and stand types. Specifically, in the 0–20 cm layer, CL had significantly higher ratios; in the 20–40 cm layer, LF and mixed forests had higher ratios; and in the 40–60 cm layer, mixed forests had higher ratios (*p* < 0.05).

**Table 3:** Stoichiometric characteristics of soil microbial biomass among different forest types.

Index	Forest types	Soil layers(cm)			
		0-20	20-40	40-60	0-60
MBC/MBN	CJ	23.25±1.65Ba	26.49±2.27Ba	27.91±2.49Ba	25.89±1.26B
	LF	22.84±2.03Ba	20.88±1.49Ca	25.90±1.88Ba	23.21±1.07B
	CL	32.77±2.87Aa	37.85±1.61Aa	41.18±5.25Aa	37.26±2.09A
MBC/MBP	CJ	31.35±2.28Aa	23.37±0.88Bb	27.72±1.61Bab	27.48±1.10B
	LF	35.56±2.74Aa	28.78±1.64Bb	25.12±1.45Bb	29.82±1.35B
	CL	35.92±3.67Ab	52.35±5.85Aab	55.99±8.77Aa	48.09±3.90A
MBN/MBP	CJ	1.38±0.09Aa	0.99±0.12Bb	1.07±0.10Bb	1.14±0.06B
	LF	1.58±0.07Aa	1.40±0.05Ab	0.99±0.04Bc	1.32±0.05A
	CL	1.13±0.09Ba	1.35±0.10Aa	1.32±0.08Aa	1.27±0.05AB

qMBC, qMBN and qMBP are the abbreviation for soil microbial entropy C, microbial entropy N and microbial entropy P, respectively.

### 3.2 Soil microbial entropy and stoichiometric imbalance across different stand types

The ranges of soil qMBC, qMBN, and qMBP were 10.97–19.18%, 9.76–17.59%, and 16.88–33.51%, respectively (**Table 4**). The aggregated data (0–60 cm) revealed that mixed forests had a significantly higher qMBC than LF by 23.49% (*p* < 0.05). However, no significant differences in qMBC were observed among the three stand types within the individual soil layers (0–20, 20–40, and 40–60 cm). For qMBN, the 0–60 cm data showed that CJ exhibited significantly higher values than LF and mixed forests (18.10% and 21.13%, respectively; *p* < 0.05). This pattern was particularly pronounced in the deep soil layer (40–60 cm). CJ and LF generally exhibited significantly higher qMBP than mixed forests (*p* < 0.05), although with variation across soil layers. In the 0–20 cm layer, mixed forests showed 21.46% higher qMBP compared than LF (*p* < 0.05); in the 20–40 cm layer, CJ had significantly higher qMBP than LF and mixed forests (*p* < 0.05), and in the 40–60 cm layer, qMBP followed the order: LF > CJ > mixed forests, with significant differences among them (*p* < 0.05).

**Table 4:** Soil microbial entropy among different forest types.

Index	Forest types	Soil layers(cm)			
		0-20	20-40	40-60	0-60
qMBC(%)	CJ	16.47±1.93Aa	18,33±1.34Aa	16.14±1.13Aa	16.98±0.86AB
	LF	17.45±1.80Aa	13.82±1.09Aab	10.97±1.21Ab	14.09±0.90B
	CL	16.72±2.56Aa	19.18±2.99Aa	16.30±2.76Aa	17.40±1.57A
qMBN(%)	CJ	14.41±0.56Ab	17.59±1.11Aa	16.17±1.10Aab	16.05±0.58A
	LF	16.22±0.77Aa	14.78±0.82Aa	9.76±0.73Bb	13.59±0.64B
	CL	13.26±1.63Aa	14.87±2.02Aa	11.61±1.33Ba	13.25±0.97B
qMBP(%)	CJ	28.61±1.69ABa	31.36±1.30Aa	24.43±2.27Bb	28.13±1.12A
	LF	27.59±1.22Ba	27.66±1.40Ba	31.22±1.99Aa	28.82±0.92A
	CL	33.51±2.60Aa	21.59±0.71Cb	16.88±0.76Cc	23.99±1.49B

Soil C/N_imb_, C/P_imb_, and N/P_imb_ ranged from 0.8–1.13, 1.36–3.04, and 1.53–3.37, respectively (**Table 5**). Aggregated data (0–60 cm) revealed that CJ and LF exhibited significantly higher values than mixed forests (23.17% and 30.49%, respectively). A similar trend was observed in the 20–40 cm layer, where LF surpassed mixed forests by 39.51% (*p* < 0.05). C/P_imb_ was significantly higher in LF than in CJ and mixed forests and N/P_imb_ was significantly higher in LF than in CJ across the 0–60 cm profile. However, layer-specific heterogeneity was evident; for example, in the 0–20 cm soil layer, the C/P_imb_ of mixed forests was significantly higher (42.29%; *p* < 0.05) than that of LF. However, the opposite trend was observed in the 20–40 and 40–60 cm layers, where the C/P_imb_ of mixed forests was 32.85% and 55.26% lower, respectively (*p* < 0.05), compared to LF. Similarly, in the 0–20 cm layer, the N/P_imb_ ratio of mixed forests was significantly higher than that of CJ and LF by 52.74% and 77.46%, respectively (*p* < 0.05). In contrast, in the 40–60 cm layer, the N/P_imb_ of mixed forests was significantly lower (by 50.15%; *p* < 0.05) than that of LF.

**Table 5:** Soil-microbial stoichiometry imbalance among different forest types.

Index	Forest types	Soil layers(cm)			
		0-20	20-40	40-60	0-60
C/N_imb_	CJ	1.02±0.13Aa	0.99±0.07ABa	1.03±0.08Aa	1.01±0.05A
	LF	1.05±0.12Aa	1.13±0.09Aa	1.02±0.13Aa	1.07±0.06A
	CL	0.85±0.07Aa	0.81±0.05Ba	0.80±0.07Aa	0.82±0.04B
C/P_imb_	CJ	1.97±0.21ABa	1.78±0.12Aab	1.50±0.07Bb	1.75±0.09B
	LF	1.75±0.17Bb	2.07±0.09Ab	3.04±0.21Aa	2.29±0.13A
	CL	2.49±0.35Aa	1.39±0.16Bb	1.36±0.18Bb	1.75±0.16B
N/P_imb_	CJ	2.01±0.13Ba	1.82±0.07Aab	1.53±0.12Bb	1.79±0.07B
	LF	1.73±0.09Bb	1.92±0.13Ab	3.37±0.31Aa	2.34±0.17A
	CL	3.07±0.52Aa	1.73±0.21Ab	1.68±0.21Bb	2.16±0.22AB

C/N_imb_, C/P_imb_ and N/P_imb_ are the abbreviation for C/N stoichiometry imbalance, C/P stoichiometry imbalance and N/P stoichiometry imbalance, respectively.

### 3.3 Correlations between soil microbial biomass/microbial quotient and soil/microbial stoichiometric characteristics

**Figure 1:**
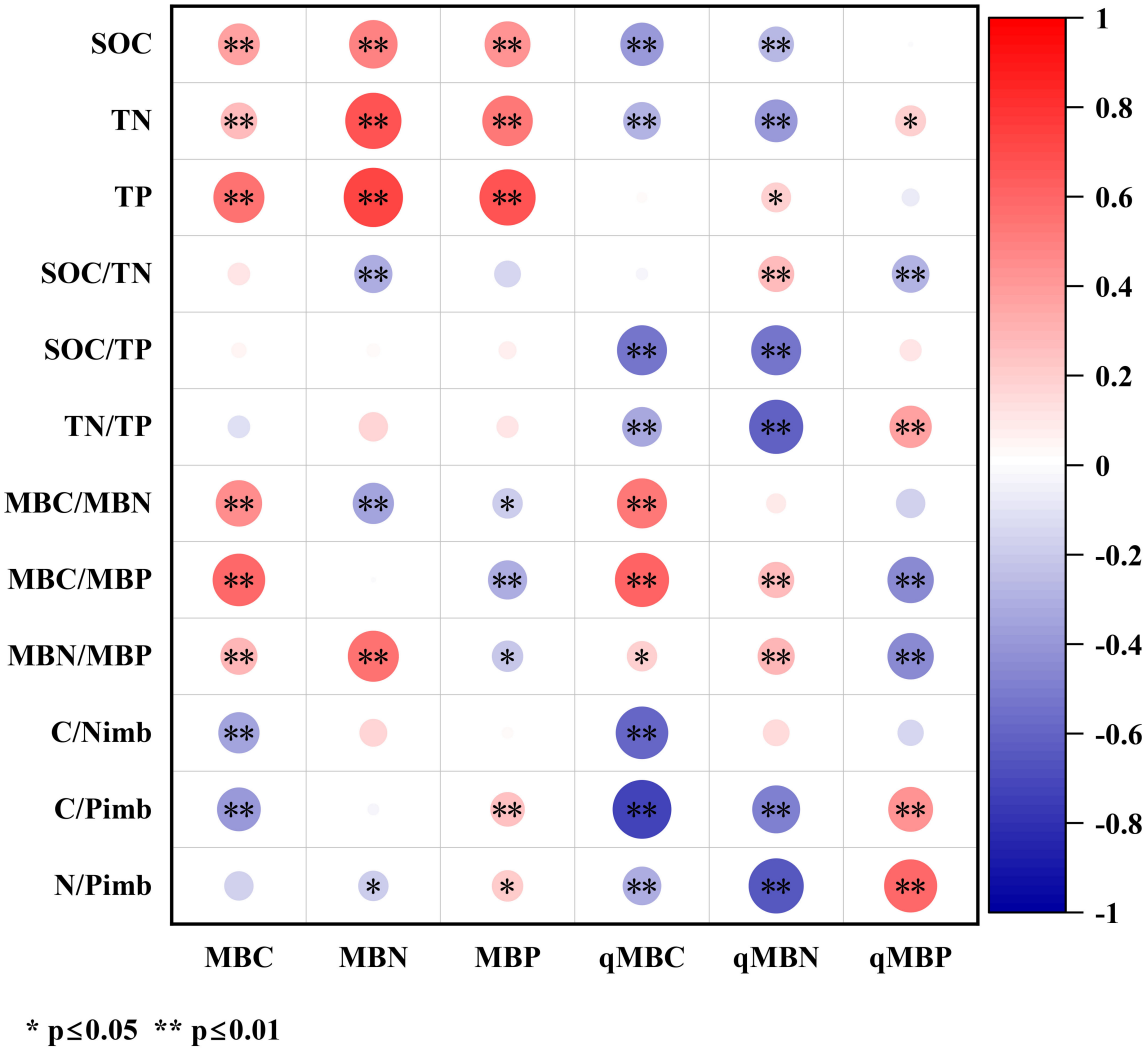
Correlation analysis between soil microbial biomass/microbial entropy and soil/microbial stoichiometric characteristics. Circles vary in color from red to blue indicating positive to negative correlations, with intensity and size showing strength. Asterisks indicate significance levels: single for p<0.05, double for p<0.01. Color scale bar ranges from -1 to 1.

As shown in **Figure 1**, soil MBC exhibited highly significant positive correlations with SOC, TN, TP, MBC/MBN, MBC/MBP, and MBN/MBP (*p* < 0.01), and highly significant negative correlations with C/N_imb_ and C/P_imb_ (*p* < 0.01). Soil MBN demonstrated highly significant positive correlations with SOC, TN, TP, and MBN/MBP but significant to highly significant negative correlations with SOC/TN, MBC/MBN, and N/P_imb_. For soil MBP, significant to highly significant positive correlations were observed with SOC, TN, TP, C/P_imb_, and N/P_imb_, whereas significant to highly significant negative correlations were observed with MBC/MBN, MBC/MBP, and MBN/MBP.

Soil qMBC showed highly significant negative correlations (*p* < 0.01) with SOC, TN, SOC/TP, TN/TP, C/N_imb_, C/P_imb_, and N/P_imb_ and significant to highly significant positive correlations with MBC/MBN, MBC/MBP, and MBN/MBP. Soil qMBN demonstrated highly significant negative correlations with SOC, TN, SOC/TP, TN/TP, C/P_imb_, and N/P_imb_ but significant positive correlations with TP, SOC/TN, MBC/MBP, and MBN/MBP. Soil qMBP displayed significant to highly significant positive correlations with TN, TN/TP, C/P_imb_, and N/P_imb_ and highly significant negative correlations with SOC/TN, MBC/MBP, and MBN/MBP.

### 3.4 Redundancy analysis of soil microbial biomass and microbial quotients in relation to soil and microbial stoichiometric characteristics

A redundancy analysis of soil microbial biomass in relation to soil and microbial stoichiometric characteristics (**Table 6**) revealed that the first and second axes explained 51.93% and 15.31% of the variation in soil microbial biomass, respectively, with a cumulative explanatory rate of 67.24%. This indicates that soil nutrients, stoichiometric ratios, microbial stoichiometric ratios, and stoichiometric imbalances effectively reflect variations in soil microbial biomass. The cumulative explanation rates for soil nutrients and stoichiometric ratios were 45.6% and 30.8%, respectively. The explanatory contributions of soil TP (*F* = 79.2; *p* = 0.002), MBC/MBP (*F* = 36.5; *p* = 0.002), MBN/MBP (*F* = 27.2; *p* = 0.002), C/N_imb_ (*F* = 16.4; *p* = 0.002), C/P_imb_ (*F* = 7.2; =0.006), and TN (*F* = 5.1; *p* = 0.03) to soil microbial biomass variation were 42.8%, 14.8%, 8.8%, 4.6%, 1.9%, and 1.3%, respectively. These parameters were identified as significant factors influencing soil microbial biomass dynamics, with soil TP demonstrating particularly dominant effects.

**Table 6:** Redundancy analysis of soil microbial biomass influenced by soil and microbial stoichiometry characteristics.

Soil and microbial stoichiometry indicators	Explains %	pseudo-F	P	RDA ordination figure
TP	42.8	79.2	0.002	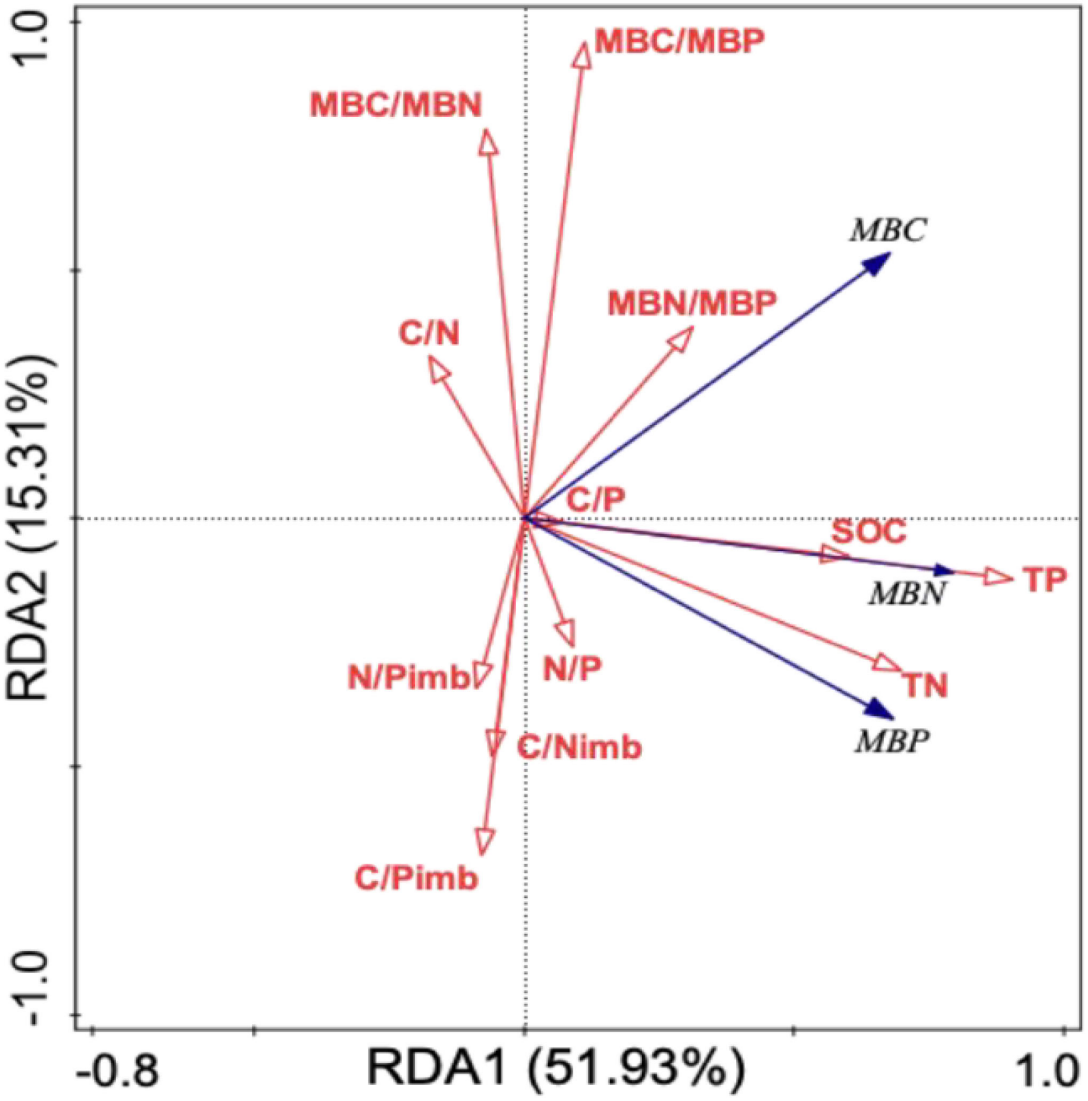
MBC/MBP	14.8	36.5	0.002	
MBN/MBP	8.8	27.2	0.002	
C/Nimb	4.6	16.4	0.002	
C/Pimb	1.9	7.2	0.006	
TN	1.3	5.1	0.03	
SOC	0.9	3.5	0.064	
N/Pimb	0.6	2.5	0.106	
C/N	0.4	1.5	0.216	
N/P	0.2	0.6	0.426	
MBC/MBN	0.1	0.4	0.528	
C/P	<0.1	<0.1	0.894	

The blue arrow represents the response variable; The red arrow represents the explanatory variable; The length of the arrow indicates the degree of influence; The angle between the arrows is the degree of correlation in table 6 and 7.

The redundancy analysis of soil microbial entropy in relation to soil and microbial stoichiometric characteristics (**Table 7**) revealed that the first and second principal component axes explained 40.54% and 15.84% of the variation in soil microbial entropy, respectively. Soil nutrients and stoichiometric ratios collectively accounted for 8.8% of the explained variation, and microbial stoichiometric ratios and imbalances showed a cumulative explanatory power of 55.3%. Among these factors, C/P_imb_ (*F* = 48.7; *p* = 0.002), C/N_imb_ (*F* = 27.5; *p* = 0.002), MBC/MBP (*F* = 13; *p* = 0.002), C/P (*F* = 11; *p* = 0.004), and MBN/MBP (*F* = 8.1; *p* = 0.008) were identified as significant determinants of soil microbial quotients, with explanatory contributions of 31.5%, 14.2%, 5.5%, 5.2%, and 3.0%, respectively.

**Table 7:** Redundancy analysis of soil microbial entropy influenced by soil and microbial stoichiometry characteristics.

Soil and microbial stoichiometry indicators	Explains %	pseudo-*F*	*P*	RDA ordination figure
C/Pimb	31.5	48.7	0.002	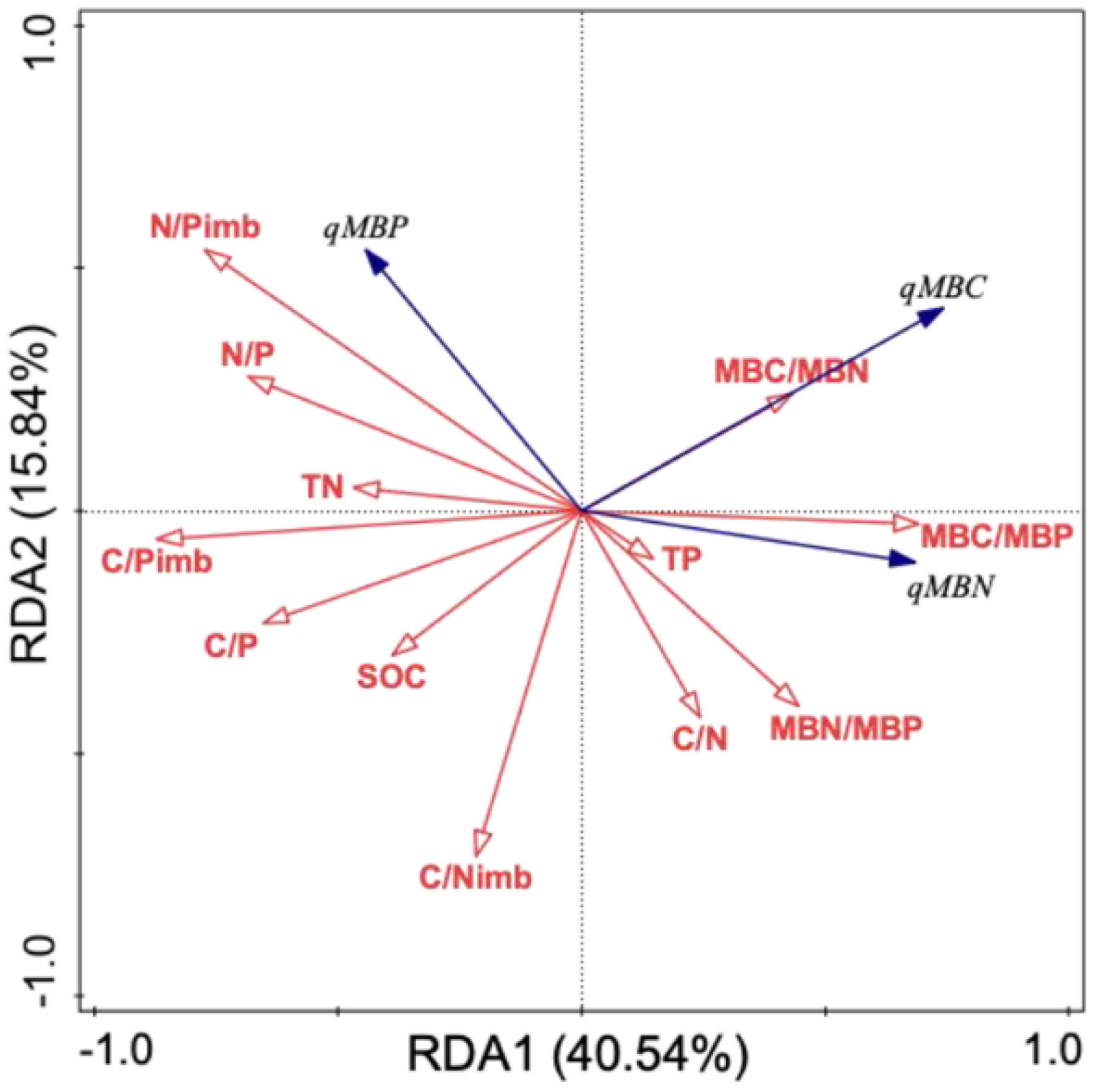
C/Nimb	14.2	27.5	0.002	
C/P	5.2	11	0.004	
MBC/MBP	5.5	13	0.002	
TP	1.4	3.3	0.084	
N/Pimb	1.1	2.8	0.098	
TN	0.9	2.2	0.132	
MBN/MBP	3	8.1	0.008	
C/N	1.1	3	0.072	
N/P	0.2	0.5	0.506	
MBC/MBN	<0.1	0.3	0.626	
SOC	<0.1	<0.1	0.858	

## 4 Discussion

### 4.1 The mixed forest significantly increased MBC and microbial stoichiometry ratio

As readily available nutrient pools in soils, MBC and MBN serve as critical agents for organic matter decomposition and mineralization (Brookes, 2001). Their dynamics are closely related to soil nutrient cycling and provide sensitive indicators of changes in soil fertility (Cesarz et al., 2022). Research has shown that the forest type impacts the distribution pattern of soil microbial biomass and can significantly alter MBC, MBN, and MBP (Devi and Yadava, 2006). Our 0–60 cm soil analysis demonstrated that conifer-broadleaf mixed forests exhibited significantly higher soil MBC content and storage than pure CJ and LF stands (**Table 2**; *p* < 0.05). This aligns with the observations of Yi et al. (2018) in subtropical plantation ecosystems. A possible reason for this is that mixed coniferous and broad-leaved forests have a more complex vegetation structure and richer litter resources, which can provide diverse C sources and living environments for soil microorganisms (Y. X. Wang et al., 2024). However, study demonstrated that monsoon evergreen broadleaf forests exhibited significantly higher soil MBC than conifer-broadleaf mixed forests and pure Masson pine stands (Yi et al., 2005). These contrasting findings may stem from differences in the soil characteristics, floristic composition, and climatic conditions between our studies(H. D. Wang et al., 2024). Our study also indicated that MBN content was relatively high in the 0–20 and 20–40 cm soil layers of the broad-leaved forest (**Table 2**; *p* < 0.05), which is consistent with the findings of previous study (Li et al., 2020). This may be attributed to the abundant N sources in the broad-leaved forest (such as root exudates and N-rich litter) and the higher N fixation capacity of the microbial community, further increasing the N content in the soil (Han et al., 2025).

Soil C/N/P stoichiometry reflects the balance of C, N, and P and is fundamental to soil fertility and ecosystem productivity (Liu et al., 2020). Soils with a balanced C:N:P ratio promote healthy microbial activity and efficient nutrient cycling, which are essential for plant growth and health (Lasota et al., 2021). Xu et al. (2014) reported that the average soil microbial biomass C:N:P was 42:6:1. In the present study, the soil microbial biomass C/N/P was 26.7:1.1:1 in coniferous forests, 29.5:1.3:1 in broad-leaved forests, and 41:1.1:1 in mixed forests, among which MBC/MBP and MBN/MBP were lower than the global average and MBC/MBN was higher than the global average. Determining whether an organism is homeostatic or non-homeostatic is an important issue in ecological stoichiometry. If an organism maintains a certain C: N: P ratio regardless of the chemical composition of surrounding resources, it is homeostatic; Nonhomeostatic organisms will adjust the C: N: P ratio with changes in the composition of resource elements (Sterner and Elser, 2002). In the fragile Karst ecosystem, soil microbial homeostasis is not strong. Other studies also show stoichiometric variability in the soil microbial biomass (Heuck et al., 2015). Unlike our research findings, Cleveland and Liptzin found an average MBC/MBN ratio of 8.6 based on extensive soil microbial biomass stoichiometry (Cleveland and Liptzin, 2007). The ratio range of MBC/MBN after afforestation in typical karst ecological areas of Guangxi Province, China is also between 6.5 and 8.1 (Hu et al., 2016). The higher MBC/MBN in our study may be due to the high SOM content of soil organic matter in the study area, whereas the relative lack of N and P resulted in low soil microbial N and P, resulting in a high MBC/MBN ratio (X. L. Wu et al., 2019). In N- and P-constrained environments, microorganisms adjust their biomass stoichiometric ratios to adapt to resource constraints (Cleveland and Liptzin, 2007). The MBC/MBN ratio reflects the main components of the soil microbial community, and its values were dominated by bacteria at 5:1, actinomycetes at 6:1, and fungi at 10:1 (Zhang et al., 2025). In this study, the MBC/MBN ratio ranged from 20.88–41.18, much higher than 10:1, indicating that fungi maybe affect the microbial biomass C and N cycle in this region, and the soil of these three vegetation types may be in a state of N and P limitation (X. L. Wu et al., 2019). Our study also found that the stand type and had significant effects on MBC/MBN, MBC/MBP, and MBN/MBP (**Table 3**), which is consistent with the conclusions of Tian (Tian et al., 2023) and Cleveland (Cleveland and Liptzin, 2007). Different tree species may lead to changes in the quantity and quality of litter or the main composition of microbial communities, whereas changes in soil microbial community composition may lead to changes in the ecostoichiometric characteristics of soil microbial biomass, especially in P-deficient soils (Heuck et al., 2015).

### 4.2 Mixed forests increased qMBC, but microbial stoichiometric imbalances were most pronounced in broad-leaved forest

As a comprehensive parameter for evaluating the sequestration efficiency of soil microbial organic C, the numerical characteristics of soil microbial entropy can characterize the efficiency level of the transformation process of organic matter to microbial mass (Tian et al., 2020). Its change is controlled by the synergistic regulation of multiple factors, such as microbial community composition characteristics, regional climate factors, and soil physical and chemical parameters (Li et al., 2024), among which key factors, such as microbial community diversity, matching degree of hydrothermal conditions, and soil C/N ratio, constitute the main driving factors (Chi et al., 2023). In this study, stand type had a significant effect on qMBC, qMBN, and qMBP (0–60 cm), qMBC had significant advantages in mixed forests (**Table 4**). The increase of qMBC in mixed forests may be related to changes in soil microbial community structure and diversity. Fungi have higher substrate utilization efficiency and lower metabolic quotient than bacteria (Floriani et al., 2024). After the transformation of forest stand types, the soil microbial community structure may shift towards higher relative abundance of fungi (Allison et al., 2007), leading to an increase in qMBC。Our research also showed that the qMBN of coniferous forest was significantly higher than that of broad-leaved forest and mixed forest in the 40–60 cm soil layer (**Table 4**). Our findings differ from those of Wu et al. (2016), who observed significant differences in qMBN between different vegetation types in the 0–40 cm soil layers. This discrepancy may be due to the differences in N availability gradient and microflora composition characteristics in the study samples, with spatial heterogeneity playing an important role in regulating soil biogeochemical processes (Liu et al., 2017). In the present study, qMBP showed obvious heterogeneity among the dominant stand types in the different soil layers (**Table 4**). This difference may be related to the differences in the morphology, availability, and microbial utilization strategies of P in the soil in the study area (Li et al., 2023). In the karst soil in this study area, the presence of P in various forms was affected by soil pH, iron and aluminum oxides, and other factors, which hindered the utilization of P by microorganisms (Qian et al., 2022).

As an ecological indicator to characterize the difference between microbial demand and substrate supply, the stoichiometric imbalance of soil and microorganisms can be used as a diagnostic parameter to analyze the nutrient coupling mechanism of the soil-microbial system (Zhou and Wang, 2016). Global-scale studies have shown that the mean values of soil microbial C/N/P imbalances are 2, 7, and 3, respectively (Mooshammer et al., 2014). In this study, the average values of soil microbial C/N/P imbalance were 0.97, 1.93, and 2.1, respectively, lower than the global average, indicating that the effectiveness of the soil substrate in the study area was higher and the microbial assimilation efficiency was better. Xiao et al. (2025) found that the average C/N_imb_ and C/P_imb_ in *Cunninghamia lanceolata* forest were significantly higher than those of broad-leaved and mixed forests, whereas the average N/P_imb_ ratio of evergreen broad-leaved forest and *Cunninghamia lanceolata* forest was significantly higher than that of mixed forest, indicating that the soil quality of *Cunninghamia lanceolata* forest was lower than that of other vegetation types. However, the present study showed that C/N_imb_, C/P_imb,_ and N/P_imb_ had higher ratios in broad-leaved forests (**Table 5**), suggesting that the soil nutrient cycling of coniferous and mixed forests in the study area was somewhat stable, whereas the soil quality of broad-leaved forests was worse than that of the other vegetation types (Mooshammer et al., 2014). This may be because litter in broad-leaved forests is relatively easy to decompose and can quickly release a large amount of nutrients; thus, soil microorganisms can obtain abundant nutrients in a short period of time (Pang et al., 2022). However, the proportion of C, N, and P released in litter does not match the needs of microorganisms, resulting in an increase in the imbalance between soil and microorganisms in the use of C, N, and P (Zhou and Wang, 2016).

### 4.3 TP and C/P_imb_ are the main factors affecting soil microbial biomass and microbial entropy, respectively

In this study, there was a significant positive correlation between soil MBC, MBN, MBP and SOC, TN, and TP content (**Figure 1**), which is consistent with the results of previous studies (Allen and Schlesinger, 2004; Wang et al., 2015), indicating that (Kästner et al., 2021). In addition, we found that TP was the main controlling factor affecting the change in soil microbial biomass in soil nutrients, with an explanation rate of 42.8% (**Table 6**). This may be attributed to the fact that P directly affects the growth rate, metabolic activity, and community structure of microorganisms (Hu et al., 2024). When the P in the soil is sufficient, microorganisms can better perform energy metabolism, nucleic acid synthesis, and other life activities, thereby promoting the growth and reproduction of microorganisms and increasing soil microbial biomass (Li et al., 2022). Previous study on karst peaks and depressions showed that there was no significant correlation between soil MBC, MBN, MBP, and TP (Huang et al., 2022), which was inconsistent with our results. This may be due to differences in the soil physical and chemical properties (such as soil moisture, pH, and salinity), vegetation types, and land use patterns in the study areas, which impact the relationship between TP and microbial biomass (Yan et al., 2010).

Consistent with the conclusion of Zhou and Wang (2016), there were significant negative correlations between soil qMBC and qMBN and SOC, TN, C/P, and N/P (**Figure 1**). The internal mechanism of this negative correlation may be due to the dynamic response strategy of microbial metabolic activities to the coordinated supply of substrate resource elements. When the system has high C/N and C/P characteristics, the microbial community is limited by the supply threshold of N and P, which directly leads to a significant decrease in microbial entropy (qMBC and qMBN)(S. Y. Wang et al., 2024). In this study, C/P_imb_ and C/N_imb_ were found to be the core regulatory parameters affecting microbial entropy variation, with explanatory rates of 31.5% and 14.2%, respectively. This conclusion is theoretically corroborated by Zhang’s research on the utilization efficiency of microbial elements, which revealed that the elemental coupling state of the soil-microbial system profoundly affects the material cycle pattern at the ecosystem level by regulating the metabolic pathways of decomposers (G. Zhang et al., 2023).

## 5 Conclusion

This study focused on the soils of three forest stand types (pure *Cryptomeria fortunei* forest, pure *Liquidambar formosana* forest, and mixed *Cryptomeria fortunei-Liquidambar formosana* forest) in the Zhazuo Forest Farm, Guizhou Province, China. It investigated the variations in soil microbial biomass, microbial entropy, and stoichiometric characteristics and analyzed the relationships between soil microbial biomass, microbial entropy, and the stoichiometric characteristics of soil and microorganisms. The main findings were as follows: (1) compared to pure forests, mixed forests exhibited significantly increased MBC, MBC/SOC, MBC/MBN, MBC/MBP, and qMBC. However, microbial stoichiometric imbalances (C/N_imb_, C/P_imb,_ and N/P_imb_) were most pronounced in the pure *Liquidambar formosana* forest, whereas qMBN and qMBP were highest in the pure *Cryptomeria fortunei* forest. (2) MBC, MBN, and MBP showed highly significant positive correlations with SOC, TN, and TP (*p* < 0.01), whereas qMBC and qMBN exhibited highly significant negative correlations with SOC and TP content (*p* < 0.01). (3) The primary factors influencing the soil microbial biomass were TP and MBC/MBP, whereas the key factors affecting the soil microbial quotient were mainly C/P_imb_ and C/N_imb_. Based on the research findings of the article, it is recommended to promote the planting mode of mixed forests in karst areas.

This study revealed the differences of soil microbial biomass and stoichiometry among different forest stands, but their microscopic mechanisms are still unclear. In the future, high-throughput sequencing technology needs to be used first to analyze the structure and key functional groups of bacterial and fungal communities, clarify the specific driving microbial communities for the increase in MBC, qMBC, and stoichiometry in mixed forests, as well as the community causes of stoichiometry imbalance. Secondly, the activity of enzymes related to carbon, nitrogen, and phosphorus cycling and their stoichiometric ratios should be systematically measured to verify the pathways through which C/P_imb_ and C/N_imb_ affect microbial biomass.

## Data Availability

The original contributions presented in the study are included in the article/Supplementary Material. Further inquiries can be directed to the corresponding author/s.
